# Are null segregants new combinations of heritable material and should they be regulated?

**DOI:** 10.3389/fgeed.2022.1064103

**Published:** 2023-01-10

**Authors:** Jack A. Heinemann, Katrin Clark, Tessa C. Hiscox, Andrew W. McCabe, Sarah Z. Agapito-Tenfen

**Affiliations:** ^1^ Centre for Integrated Research in Biosafety and School of Biological Sciences, University of Canterbury, Christchurch, New Zealand; ^2^ Climate and Environment Division, NORCE Norwegian Research Centre AS, Tromsø, Norway

**Keywords:** risk assessment, regulation, scale, gene technology, null/negative segregants, sustainable development goal 12, sustainable development goal 13

## Abstract

Through genome editing and other techniques of gene technology, it is possible to create a class of organism called null segregants. These genetically modified organisms (GMOs) are products of gene technology but are argued to have no lingering vestige of the technology after the segregation of chromosomes or deletion of insertions. From that viewpoint regulations are redundant because any unique potential for the use of gene technology to cause harm has also been removed. We tackle this question of international interest by reviewing the early history of the purpose of gene technology regulation. The active ingredients of techniques used for guided mutagenesis, e.g., site-directed nucleases, such as CRISPR/Cas, are promoted for having a lower potential per reaction to create a hazard. However, others see this as a desirable industrial property of the reagents that will lead to genome editing being used more and nullifying the promised hazard mitigation. The contest between views revolves around whether regulations could alter the risks in the responsible use of gene technology. We conclude that gene technology, even when used to make null segregants, has characteristics that make regulation a reasonable option for mitigating potential harm. Those characteristics are that it allows people to create more harm faster, even if it creates benefits as well; the potential for harm increases with increased use of the technique, but safety does not; and regulations can control harm scaling.

## Introduction

Genetic modification is a continuum of interventions that have led to the more efficient selection of desired, and sometimes undesirable, organisms. Gene technology is different from this. Technology is a continuum of advances in tools and practices that accelerate modifications in genes at will that result in efficiencies that produce more changes in more species, genes, and traits but in less time. As described by the United States National Research Council in 1989, ‘biologists now have the means, by directly modifying genes, to alter living organisms more quickly and more precisely than has been done by nature and humans over millennia’ (NRC, 1989).

The efficiency of making and isolating desired genetic variants using the accumulating tools and practices of gene technology reached a tipping point in the 1970s (Singer, 2002). It was then that gene technology became an object of governance through regulations.

The work described in this paper was inspired by an observation made in a meeting sponsored by the United Nations Food and Agriculture Organisation. The observation was that for many countries, the phrase ‘new combination of heritable/genetic material’ triggers the regulation of genetically modified organisms (McMurdy, 2021). The utility of this phrase has consistently been challenged since it first appeared almost 50 years ago. Genome editing has re-invigorated these challenges.

In part 1, we trace the history of the phrase. It is commonly found in legislations, although not used by all countries, that regulate genetically modified organisms (GMOs). The phrase appears in the Cartagena Protocol on Biosafety, a sub-protocol of the Convention on Biological Diversity (CBD, 1992; CBD, 2003). This trigger phrase is thus the closest to an international consensus mechanism in gene technology governance.

Why was *this* phrase chosen? It appears to be a phrase that proponents of deregulation of some or all techniques of gene technology find problematic. Yet, it persists. What utility does it have? What is the future without it?

The priority audiences for our work are policymakers and regulators. The intention is to use insights from the history of this phrase to explore how it applies to the techniques of gene technology. Unpacking this history of thought hopefully will be of general use to the science and technology communities as well.

In part 2, we discuss legislative scope. Inclusion criteria and definitions determine what legislation covers. Some things are excluded from the scope because they do not qualify under the inclusion criteria or are actively defined as exclusions. Some things are included and then exempted. Insights into how risk is understood and managed explain this convoluted approach.

Legislation has purpose. For governing gene technology, the purpose is ‘to protect human health and the environment from the possible adverse effects of the products of modern biotechnology” (CBD, 2003). What is defined as a GMO should be broad and flexible enough to achieve the protection of human health and the environment.

A null segregant is one type of outcome of a production line using genome editing on plants. In some places, these products are being excluded from the scope of GMO laws. They are the most stringent challenge we could think of for testing whether regulations with a ‘new combinations’ trigger were still fit for purpose.

In part 3, we describe the hazards to be managed. The decision to exclude or exempt null segregants should depend on whether their continued regulation would contribute to the protection of human health and the environment.

There are many kinds of harm that could, and should, be considered, ranging from biological to socioeconomic. The biology exists in environmental and socioeconomic contexts. Some risks can only be seen where these overlap with each other. We limit ourselves mostly to the biological dimension, as might be revealed through a scientific risk assessment, but are cognisant of other dimensions and occasionally will refer to these.

Two predominant perspectives of a scientific risk, which raise conflict over the interpretation of the phrase, will be discussed in part 4. Again, these are tested in their extremes by null segregants.

So, it is time to introduce null segregants (sometimes called negative segregants). In this context, they are organisms that are living derivatives of genetically modified organisms (for extended discussion, see Box 1). The implication is that ‘segregants’ have been rebuilt into organisms that are not genetically modified. Alluding to Schrödinger’s cat, null segregants are GMOs and not GMOs at the same time, placing such organisms at the heart of current debates on regulation.

A common kind of plant null segregant is a descendant of a parent that has held a DNA molecule used to support the transcription of a genome editing nuclease such as Cas9. For our discussion, a null segregant can be a descendent of a cell that has experienced the insertion of a nucleic acid, as explained in the following:

Several strategies have been developed to remove or prevent the integration of gene editor constructs, which can be divided into three major categories: 1) elimination of transgenic sequences *via* genetic segregation; 2) transient editor expression from DNA vectors; and 3) DNA-independent editor delivery, including RNA or pre-assembled Cas9 protein-gRNA ribonucleoproteins (RNPs) (Gu, Liu, and Zhang, 2021).

The discussion on regulations is relevant beyond this special case. It will apply to any intended deletion using the techniques of genome editing (for examples, see van de Wiel et al., 2017) or other methods, including the use of recombinases (Ow, 2007; Sukegawa et al., 2021).

BOX 1 What are null segregants and how are they used? The term ‘null segregant’ is often used as a synonym for ‘negative segregant’ or ‘trait-negative segregant’. The common element in different definitions of null or negative segregants is that they are derived from GMOs (Eckerstorfer et al., 2019a). The Academy of Science of South Africa says that ‘Null, or negative, segregants refer to the non-GMO offspring in a breeding programme where GM elements or traits were used transiently’ (ASSAf, 2016).

The European Food Safety Authority (EFSA) describes a null/negative segregant this way: ‘Plants that are negative segregants lack the transgenic event and can be produced, for example, by self-fertilisation of hemizygous GM plants, or from crosses between hemizygous GM plants and non-GM plants’ (EFSA, 2011). EFSA stops short of predetermining that the null segregant is a non-GMO. Others also limit the reference to non-transgenic rather than non-GMO, ‘i.e., they no longer contain the inserted transgenes’ (Eckerstorfer et al., 2019a).

The rationale behind calls to deregulate null segregants is that the organisms have been modified, but the organism contains ‘no genetic modifications’ (Camacho et al., 2014). In the context of the Academy of Science of South Africa opinion, a GMO is an organism that has a new piece of DNA inserted into it, e.g., a ‘transgenic event’ or ‘transient event.’ Therefore, a null segregant is not a GMO because it has lost the insertion.

The phrase ‘new combinations of genetic material’ is problematic for those who subscribe to a view of GMOs as being only those organisms that contain the DNA inserted into them because it does not obviously place null segregants out of the regulatory scope. They have, as part of the process of manufacturing, a deletion rather than an insertion, resulting in a new combination of genetic material.

In the following paragraphs, we will provide a short description of some uses, illustrating where a null segregant emerges in the process. As observed by others, these strategies can be combined in various ways, including by serial application at the same or multiple genes in the development of a product (Sukegawaet al., 2021).

Example: accelerated (rapid cycle) breeding.

Shortening the juvenile stage of a plant reduces the generation time. This could be particularly useful in long-lived species, such as fruit trees. A transgenic approach is to over-express genes that induce flowering in younger trees. A null segregant arises from offspring when one parent was hemizygous for the transgene (ASSAf, 2016).

Example: reverse breeding.

Crossing two elite plant varieties together sometimes produces offspring (F1) with superior traits. To preserve the benefits of heterozygous loci, called heterosis, recombination between sister chromosomes must be prevented.

One parent of the desired F1 is genetically engineered by the insertion of a transgene that stops the production of an enzyme needed for recombination between sister chromosomes during the production of gametes. F1 are produced by crossing this parent with another elite variety. After this step, haploid cells from the F1 are induced to duplicate their post-meiotic haploid genomes (‘doubled haploidy’ Lenaerts, Collard, and Demont, 2019). These plants can be crossed to indefinitely perpetuate the desired F1 phenotype of the founder (Wijnker et al., 2012).

In this case, only one (grand)parent has a copy of the transgene, and the gene is dominant. If non-transgenic offspring are desired, then they could be separated from those with the transgene by various means. Those offspring would be null segregants.

Example: biased mutagenesis with segregation.

Modified site-directed nucleases remove an amine group from a cytosine or adenine nucleotide at a target sequence without breaking the phosphodiester bonds that hold a strand of DNA together (Wada et al., 2020). A dCas9 fusion to a C or A deaminase can be used to create point mutations (C to T or A to G). A cytidine deamination was used to introduce a mutation in wheat that conferred multiple herbicide resistance (Zhang et al., 2019).

Offspring without the gene for the nuclease or its guide arise either by the loss of the transgene(s) or segregation of the chromosome during meiosis (Wada et al., 2020).

Example: large deletions.

Plant genomes can be complex because of redundancy and polyploidy. In order to achieve a new trait through gene elimination, it can be necessary to simultaneously mutagenize multiple loci or to remove the large tracts of DNA (Duan et al., 2021). Concurrent multi-locus editing and large deletions was demonstrated in soybean. The researchers modified eight different loci and generated deletions up to 1 Mb.

Notably, in all examples, the transgene, in whole or part, may still find its way into plants expected to be null segregants, and thus continued confirmation would be needed to meet the exclusion or exemption criteria.

## Origins, present status, and interpretations of ‘new combinations’

The phrase novel or ‘new combinations of heritable/genetic material’ has been in GMO regulations since the 1970s. We believe that the phrase may have first appeared in the UK Health and Safety regulations in 1978.

Search terms used in Google Scholar and/or Google were ‘new combinations of heritable/genetic material’, ‘Genetic Manipulation Advisory Committee’, “self-cloning’, ‘recombinant DNA’, ‘oligonucleotide’, and ‘gene/genome editing’, with an emphasis on publications before 1985. News articles in scientific publications and publications in legal and sociological journals were included. We retained those materials wherein relevant techniques, development of regulations, or interpretations of regulations were discussed.

The phrase is homologous to that used in both the Aarhus Convention (UNECE, 2021) and Directive 2001/18/EC of the European Union (Tooze, 1980-1981). It survives in the Cartagena Protocol on Biosafety of the United Nations Convention on Biological Diversity which governs the movements of living (genetically) modified organisms (Table 1).

**TABLE 1:** Phylogeny of regulatory trigger phrases.

Phrase	Source
2.-(1) In these Regulations, unless the context otherwise requires ‘genetic manipulation’ means the formation of new combinations of heritable material by the insertion of nucleic acid molecules, produced by whatever means outside the cell, into any virus, bacterial plasmid, or other vector system so as to allow their incorporation into a host organism in which they do not naturally occur but in which they are capable of continued propagation	United Kingdom (UK, 1978)
Techniques of genetic modification referred to in Article 2(2)(a) are inter alia: 1) Recombinant nucleic acid techniques involving the formation of new combinations of genetic material by the insertion of nucleic acid molecules produced by whatever means outside an organism, into any virus, bacterial plasmid or other vector system and their incorporation into a host organism in which they do not naturally occur but in which they are capable of continued propagation	Directive 2001/18/EC of the European Union (Annex 1A) (EU, 2001)
‘Living modified organism’ means any living organism that possesses a novel combination of genetic material obtained through the use of modern biotechnology	Article 3(g) of the Cartagena Protocol on Biosafety to the United Nations Convention on Biological Diversity, which governs the movements of living (genetically) modified organisms (CBD, 2003)

Commentators and objectors voiced their concern about the draft regulation of the UK Health and Safety regulations in early 1979 (Bodmer et al., 1979). The controversy was summarized in the following passage of a *Nature* news article:

The chief difficulty, says Dr. Sherratt, arises in the meaning assigned to ‘they’ in the phrase ‘in which they do not naturally occur’. If it refers to the ‘new combinations’, then all self-cloning experiments are included in the guidelines. If, on the other hand, it refers to the ‘nucleic acid molecules’, as most scientists have assumed it does, then all self-cloning experiments are exempt from regulation. This is the point that GMAG [Genetic Manipulation Advisory Committee] has recently been debating (Redfearn, 1979).

Self-cloning was defined by example as ‘cloning *E. coli* genes into *E. coli* organisms’, that is, no insertion of foreign DNA into a recipient organism. Self-cloning overlaps with the present concepts of cis- and intragenics. A few months later, self-cloning experiments were confirmed to be within the scope of the regulations (Anonymous, 1979).

The important point about this historical anecdote is that the reference of the pronoun ‘they’ in the phrase was to ‘new combinations’. The following is how the regulation looks when the pronoun is replaced with a repetition of the intended reference:

2.-1) In these Regulations, unless the context otherwise requires-‘genetic manipulation’ means the formation of new combinations of heritable material by the insertion of nucleic acid molecules, produced by whatever means outside the cell, into any virus, bacterial plasmid, or other vector system so as to allow their incorporation into a host organism in which new combinations of heritable material (made as described) do not naturally occur but in which new combinations of heritable material (made as described) are capable of continued propagation.

The phrasing ‘incorporation into a host organism’ covered what was then almost exclusively the use of plasmids that were not inserted into the chromosome of engineered bacteria (Berg et al., 1974). It was broad enough to remain valid for what was yet to become the mainstay for plant and animal transformations that require insertion into the chromosome. It also anticipates transient expression using DNA or RNA molecules that do not replicate.

The aforementioned should be no surprise because some techniques of genome editing, such as oligonucleotide mutagenesis, were already in use while these regulations were being drafted (Heinemann et al., 2021). In addition, other site-targeting techniques were being used in plants in the 1990s, such as Cre recombinase in the development of the high lysine maize line LY038. These too were expected to not only increase the rate at which traits could be stacked and be optimally expressed but also to create null segregants of superfluous antibiotic resistance genes (Ow, 2007). The intended interpretation of the phrase was, thus, both relevant in earlier decades and, in effect, ready for plant genome editing.

Regulators responded to objectors by maintaining this phrase within a risk category framework. The categories were aligned with physical (infrastructure) or biological approaches to contain organisms with new combinations of heritable material. Containment measures varied according to the severity of risk to human health or the environment. ‘Differentiated risk categories’ based on the severity of risk was an idea promoted by Sydney Brenner (GMAG, 1978) who recommended it originally to the Ashby Working Party (Brenner, 1974b). A similar recommendation was later arrived at by a ‘consensus’, defined as the large-fraction majority attending the 1975 Asilomar conference.

The ‘new combinations’ phrase maintained attention on the presumed ultimate hazard that might be produced using gene technology. The risk category policy provided proportional responses. This sometimes moved the conflict to disagreements over proportionality and, further down the road, to the affordability or convenience of practice (Norman, 1975; Novick, 1977; Wade, 1977; Pritchard, 1978; Bradford et al., 2005).

## Legislative scope

An organism is regulated as a GMO if it is defined as a GMO or it is not exempted from GMO regulations. What is a GMO and what is not a GMO is determined by inclusion and exclusion criteria (Jenkins et al., 2021). For example, the inclusion criteria of the European Union Directive 2001/18/EC are organisms wherein ‘the genetic material has been altered in a way that does not occur naturally by mating and/or natural recombination’ (EU, 2001).

Unlike the EU, the United States did not introduce legislation specific to GMOs (Camacho et al., 2014). They introduced a Coordinated Framework for Regulation of Biotechnology instead (USDA, 1986). This framework made governance decisions a regulatory discretion for the Food and Drug Administration, Environmental Protection Agency, and the Department of Agriculture (Jenkins et al., 2021) within the context of ‘[e]xisting statutes [which] provide a basic network of agency jurisdiction over both research and products’ (USDA, 1986). These underlying legislative instruments particular to each regulator define the mandate which in turn informs the policy that defines inclusion criteria.

The EU Directive excludes humans and organisms with altered genetic material because of spontaneous events outside of human interventions. Annex 1 A Part 2 of the EU Directive contains a list of specific techniques that do not create GMOs.

Annex 1 B of the EU Directive provides a list of processes that create GMOs. Organisms created using any of these ways are then exempted from the scope of the regulations. In other words, GMOs created using these processes are not subject to the specific regulatory requirements of Directive 2001/18/EC. They may still be subject to regulation but not by the Directive. Similarly, if one US agency excludes an organism from consideration under its policy, then that same organism may be regulated by a different agency or by none of them.

Deregulation of something that was previously regulated can be achieved by defining it out of scope (exclusion) or by defining it out of regulations (exemption). Similarly, deregulation of certain ways to alter genetic material unnaturally may be carried out either by excluding those specific ways from the definition of a GMO or by exempting them from the regulations.

Deregulated products must meet the exclusion or exemption criteria. The standards of evidence and the expectations of reporting are not always specified by legislation. Australia recently excluded from scope new genomic/breeding techniques (NG/BTs) that create ‘organisms modified by repair of single-strand or double-strand breaks of genomic DNA induced by site-directed nuclease, if a nucleic acid template was not added to guide repair’ (Jenkins et al., 2021). This is a process that may also be used in the development of null segregants. Australia delegates responsibility for meeting the exclusion criteria to product developers.

The burden of proof may be set high, but if there is little chance of being called to account, then the expectations become *pro forma*. These concerns were raised by Fonterra, New Zealand’s biggest dairy company, during a consultation with Food Standards Australia New Zealand (FSANZ). Fonterra observed that

Clear guidance and objective criteria are needed on which to base and define an appropriate term to express ‘same’, ‘similar’, or ‘equivalent’. This is to ensure risk assessment for NBTs is effective and captures NBT food that may pose a greater risk compared to conventional food, particularly where changes may be heritable (Fonterra, 2021).

The company supported the proposed deregulation of null segregants provided that ‘evidence exists they have not inherited a genetic modification’ because ‘[a]lthough progeny are selected that have not inherited any new DNA and do not display the GM trait, it is unclear whether there could be other unintended outcomes…Also, it may also be possible for GM progeny to be mistakenly released as null segregants’ (Fonterra, 2021).

## Are null segregants a hazard?

Risk is the (probability of harm) x (severity of harm). A technical or scientific risk assessment is carried out to estimate risk (CBD). The first step is to identify hazards. A hazard is ‘the potential of an organism to cause harm to human health and/or the environment’ (CBD, 2014). In the next step, exposure is assessed. The hazard and exposure information are integrated into an estimated likelihood and magnitude of adverse effects. Afterwards, any risks considered to be unacceptable may, following adoption of a mitigation strategy, become tolerable. A mitigation strategy may be, for instance, to eliminate a hazard or to modify the likelihood of exposure to a hazard.

Null segregants are a product of gene technology. They are the end-product of cumulative interventions. Null segregants are hazards if in some environment unintended changes or unintended outcomes of intended changes lead to adverse effects. Furthermore, as the end product of genome editing or similar techniques, intended null segregants may be uniquely eligible for utility (process-based) patent intellectual property rights. These rights help to concentrate the seed supplier market into a smaller number of companies from which farmers can choose (World Bank, 2007). The effect is to fundamentally alter the potential exposure pathways compared to the products of conventional breeding.

In the same way that genome editing reactions increase the efficiency (a term we prefer over the less precise term precision) of achieving a desired outcome in a target genome, they also increase the efficiency of reacting at other locations in the genome. The reactions are not actually between a lock and a key, but between molecules with a range of binding affinities (Kawall, Cotter, and Then, 2020). Although those reactions will be biased toward the characteristics of the target location and, therefore, less ‘random’, the secondary targets are still largely beyond our ability to comprehensively identify in advance because they are influenced by biophysical parameters at the nanoscale. Moreover, the active ingredients such as the nuclease or oligonucleotide may persist in a cell long after the cell has lost the preferred binding site. Once the primary site has been changed, the conditions shift to favour activity at secondary sites.

The issue of off-target effects has dogged gene technology since the first claims of it being more precise than other tools, including other tools of gene technology. In his 1959 Nobel lecture, Joshua Lederberg described this kind of biotechnological imaginary, calling it the ignis fatuus of genetics; ‘the specific mutagen, the reagent that would penetrate to a given gene, recognize it, and modify it in a specific way’ (Lederberg, 1959). Considerable effort has been expended to deliver on the implications of precision—that is, precision not only in a more efficient generation of intended on-target changes as in Lederberg’s description but also in the near or total absence of unintended on- and off-target changes. This has yet to be achieved.

Most of the ‘reagents’ of genome editing, such as the Cas9 family of site-directed nucleases, are very new and still being refined. The refinement process runs parallel with new discoveries that expand the awareness of what activities need refining.

In parallel, the engineering of Cas9 proteins has been performed to enhance the specificity…On the other hand, new types of mutations, which have not been addressed so far, were recently reported. Kosicki et al. observed unexpected large deletions (up to 9.5 kb) elicited as a result of Cas9-based genome editing in mammalian cells. Although such unexpected large deletions have not yet been reported in plants, the possibility of their occurrence should be taken into account (Wada et al., 2020).

Understanding what makes the so-called new techniques new is central for understanding what gene technology regulation can do to ensure their safe use. Part of the difference between when current regulations were written and now is that the tools for genome editing free the process from its dependence on a physical infrastructure. Laboratories were not created to provide physical containment for biosafety, even if they can also serve this purpose. They were needed to control contamination and protect fledgling GM cells during the engineering process, and part of this equation is a highly skilled and rate-determining workforce. By the time the process was concluded, there were a small number of candidate GMOs to evaluate for both safety and usefulness as products. The others were destroyed and discarded.

Indeed, this is the process described by the US National Research Council in its often cited 1989 report on field releases. Their Figure 2.1 shows a series of steps, all neatly confined in boxes, going from ‘biological source material→genetic modification by classical or cellular/molecular methods←→selection of desired form→evaluation in laboratory and/or field tests→large scale introduction’ (NRC, 1989).

This picture of the pipeline is a biotechnological imaginary. If this were followed, then null segregants could be proven to exist. To some standard, they could be shown to reset to their non-modified parental state.

The technology community does not share this imaginary. Our imaginary includes emancipation of the techniques from stringently controlled environments during manufacture. Active ingredients can already be embedded into new formulations that allow modification without using specialist laboratory facilities or skilled practitioners (Heinemann and Walker, 2019; Le Page, 2019). The patent literature even foresees their use in the grocery store (Heinemann and Walker, 2019). Deregulation of intended null segregants may *de facto* deregulate the organisms—e.g., bacteria, fungi, and invertebrates—exposed to the technology along the way of developing the intended null segregrant, whether that be a plant or something else. Thus, the fundamental risk to regulate not only includes any particular null segregant with its intended and possibly unintended change, but also the risk of all the failed candidates that are discarded in the process and all the presently unpredictable intended changes that might be attempted by someone in the future.

In general terms, we find support for the conclusion of the Academy of Science of South Africa (ASSAf, 2016) and the Australian decision (Jenkins et al., 2021) that regulatory triggers aligned to limit the potential harm in using gene technology (which are often called process-based) have proven to be effective and flexible. That is only possible, however, if the process is concluded with a case-by-case risk assessment.

The predetermination of risk category is decidedly different because it is a case-by-case determination of a legislative scope. Tiered (de)regulation (Bratlie et al., 2019; Everett-Hincks and Henaghan, 2019) or threshold-based triggers (ASSAf, 2016) are based on normative judgements of equivalence, as referred to by Fonterra (2021), and include arbitrary estimates of how many nucleotides must change to create a hazard. However, that kind of discretion would remove significant activities of gene technology beyond routine public accountability.

A New Zealand High Court ruling pivoted on this distinction. The world’s first legal challenge to the inclusion of genome editing techniques was presented in the High Court of New Zealand (Kershen, 2015). The regulator had issued a determination that organisms made using specified genome editing reactions did not result in a GMO for the purposes of regulation. The judge said that the regulator was making legal interpretations of scope,^1^‘while the [regulator] may determine whether an organism is within the definition or the regulation, where there is doubt about that (and in particular where different experts could come to different views) a more cautious approach is for that decision to be made *via* a regulation’ (Mallon, 2014). The regulator expressed a normative judgement about how similar those reactions were to the processes exempted by law. In doing so, the regulator made a decision about the exemption criteria rather than whether any particular organism was a GMO.

The next question is what type of risk assessment process would be proportionate. Although the question is larger than biological/technical risks alone (EGE, 2021; Engelhard et al., 2021), we limit our work here to distinguishing risk as a technological trajectory from risk as a hazard.

Deregulation of a class of GMOs such as null segregants makes some uses of gene technology unaccountable to public oversight. Null segregants do not exist until they are proven to exist by demonstration that the final organism is reset to its non-modified parental state (Buchholzer and Frommer, 2022). There have been failures to identify unintended changes in genomes even when there was motivation to do so (Windels et al., 2001; Rang, Linke, and Jansen, 2005; Regalado, 2020).

Deregulation removes verification requirements. If null segregants were deregulated, then the steps to them may be wholly or partially deregulated too. They may be amplified to high numbers and distributed under the most stringent intellectual property rights protections for which they can qualify due to the process of manufacture.

Any sincere effort to demonstrate that a null segregant was truly the outcome would be nearly as demanding if excluded or exempted from GMO regulations. Shifting the responsibility to developers to do so rather than to report to regulators that they have done so (and supply the evidence) is a way to circumvent the legislative objectives in practice. Deregulation might also (or instead) be a way to evade the democratic processes through which labelling requirements have been imposed. Labelling empowers consumer choice and the costs of market rejection that labelling makes possible are high (Addey, 2021).

## Technological risk perspectives

One perspective can be summarised this way. ‘Genetic change becomes the primary indicator and trigger for regulation – as it should because it is the root source of risk’ (ASSAf, 2016). A contrasting perspective is that risk is much more than a genetic hazard. The *root source* of risk is how technology that causes rapid and specific genetic change can cause harm.

Risk is routinely assessed and managed by a dual process-and-product approach. For example, construction of aeroplanes is regulated, and the constructed products are tested before use.

A defective aircraft is a hazard, not a risk. Risk is the probability of making a defective aircraft, multiplied by the severity of the harm it would cause if it failed. A small plane crashing may be judged as having low harm, but if deregulation results in many more planes, the probability of harm increases. Risk increases too without a change in the severity of harm an individual small plane may cause.

Combining elements of process and product is also common in gene technology regulation (Eckerstorfer et al., 2019b). For example, in the United States, which is often held up as an example of product-based GMO regulations, there is a process-and-product trigger (Wolt, Want, and Yang, 2016). ‘The USDA regulatory process for GE crops is triggered by the use of “plant pests” in any portion of the modification process or the derived potential of the GE crop to behave as a plant pest. In practice, the routine use of pest-derived genetic components triggers a *de facto* process-based regulatory regime by the USDA’s inspection branch’ (Camacho et al., 2014). Although the EU uses process-based inclusion criteria, it also assesses products (Eckerstorfer et al., 2019b).

Over the past couple of years, some countries have introduced guidelines that regulate the use of null segregants in agriculture in a way similar to conventionally bred organisms, provided that inserted nucleic acids were removed (Turnbull, Lillemo, and Hvoslef-Eide, 2021; Buchholzer and Frommer, 2022). Others have proposed to deregulate some uses of gene technology based on the number of nucleotides changed in an organism’s genome (Bratlie et al., 2019; Jones et al., 2022). In both instances, risk has been rationalised as a property of nucleic acids rather than of technology. Herein, risk is narrowly defined and hardly perceived as different from hazard.

### Risk trajectory vs. hazard control

When risk is considered along the entire technological trajectory, it is possible to envision both different sources of hazard and exposure and different strategies for risk mitigation (Pavone, Goven, and Guarino, 2011). The trajectory increases the level of analysis from the narrowly defined biological hazard to the potential to interfere with a high-level social goal (Mueller and Flachs, 2022; Heinemann and Hiscox, 2022). Mitigation options also grow from being tweaks to the technology to whether it is even among the best available options (Aga and Montenegro de Wit, 2021; EGE, 2021; Engelhard et al., 2021).

At the technical level, a trajectory is grounded in what makes gene technology a technology, rather than what makes a gene or an organism a product of a technology (Shah, Ludwig and Macnaghten, 2021). The emphasis in most discussions is on the latter; when is something ‘changed enough’ to be considered a GMO? However, the answer to this does not consider how exposures might differ between an organism developed at an industrial scale and one that arises by chance. Instead, the question could be when is it ‘safe enough’ to use gene technology?

Techniques of gene technology can be grouped, like members of a species, by shared characteristics. The distinguishing feature is *harm scaling* rather than similarity of hazards created through technological or natural processes. Continuing with the taxonomic nomenclature familiar to biologists, gene technology is the genus within the family ‘technology’. It is composed of member techniques that have three characteristics (Heinemann et al., 2021). 1) They allow people to cause more harm faster, even if it also creates benefits. 2) The potential for harm increases with more use of the technique, but safety does not. 3) Regulations can control harm scaling.

The generic value of gene technology is its scale gearing. This gearing increases with newer techniques. ‘Genome editing technologies have led to a new era of genome engineering, enabling an effective, precise, and rapid engineering of the plant genomes’ (Wada et al., 2020). Gene technology advances on this basis, with the newest of tools always being ‘simple, efficient, and cost-effective’ (Xia, Wang and Zhu, 2021) compared to what had been available earlier, further increasing its use. That is a property that makes it possible to deliver the benefits it promises at commercial time and production scales. The source of inseparable potential for harm is this same property that creates hazards at scale.

Scale-triggered regulation of gene technology unifies the management of various methodologies under a common risk genus, or technological trajectory. It provides consistency and clarity to regulations (Heinemann et al., 2021).

### Null segregants remain a hazard on the trajectory of technology

Scale increases in the production line of null segregants also increase the rate of production of both unintended outcomes from intended changes and unintended outcomes from unintended changes (Kawall et al., 2020; Antony, Hinz, and Wyrick, 2022; Chu and Agapito-Tenfen, 2022). The Asilomar scientists were aware of the risk amplification with scale. Sydney Brenner submitted that

[t]he essence is that we now have the tools to speed up biological change, and if this is carried out on a large enough scale then we can say that if anything can happen it certainly will. In this field, unlike motor car driving, accidents are self-replicating and could also be contagious (Brenner, 1974a).

Brenner had captured the raison d'être of regulation in two sentences. Monast modernises and focusses Brenner’s take onto genome editing. He says that ‘[g]ene editing speeds the process, allowing it to outpace traditional regulatory responses. Gene editing also allows a relatively small number of scientists to guide how evolution occurs. Furthermore, genetic modifications may produce different results than conventional breeding’ (Monast, 2018).

The point being that even if none of the unintended outcomes of intended changes or unintended outcomes of unintended changes prove to be harmful, the probability of harm approaches certainty if the process is sped up.

The issue of speed was also relevant in the New Zealand High Court challenge to a regulator determination that certain genome editing techniques such as the use of site-directed nucleases were sufficiently similar to chemical and radiation mutagenesis, which was on the list of exclusions, to qualify for exclusion.

The Court found compelling the view that exclusions and exemptions from governance were decisions to be made with regard to how the technology could scale. In footnote 49, the judge elaborated on this by describing the development of the governing legislation (the Hazardous Substances and New Organisms Act, HSNO Act).

Specifically, a paper from the Office of the Minister for the Environment to the Chair of the Cabinet Economic Committee… discusses the proposed regulations to be made under the Act. It says that ‘the advent of genetic manipulation has enabled a greatly increased rate and specificity of change’. It notes the need for ‘clear definitions’ as to what is or is not ‘genetic manipulation…a number of techniques have been identified which can be shown not to produce rapid or extensive change in the nature of the organism. These techniques have been identified, either on the basis of an extended record of use of the technique, or on the basis of scientific knowledge of the effect of the technique’. Similarly, an 18 May 1998 Cabinet Economic Committee Paper notes that a ‘number of techniques have been identified which can be shown not to produce rapid or extensive change in the nature of the organism. It is proposed that these techniques be formally excluded from the scope of genetic manipulation through the regulation making powers in the HSNO Act’. That paper further notes that the Minister for the Environment recommends that the Committee ‘agree to establish by regulation…the proposed thresholds for genetic manipulation that exclude from consideration under the Act genetic techniques shown not to produce rapid or specific change in the nature of the organism’ (Mallon, 2014).

## Conclusion

Reflecting on the events that motivated the formal legislative governance of gene technology, Margaret Singer observed that new ‘techniques enabled researchers to make direct changes in DNA structure to accomplish a predetermined purpose. Rather than waiting for the chance emergence of a desirable allele and then breeding it into a variety of plant, biologists can now design alleles to meet their needs’ (Singer, 2002). This in turn fuelled the scale at which such changes could and would be made.

The US National Research Council conceptualised this as a graphic titled ‘*Increase in power of genetic modification over time*’ (NRC, 1989). Paradoxically, they turned away from the regulation of this power to the regulation of putative hazards.^2^ In that era, there was no conception that the use of genome editing could include applications without controlled exposures, such as in the outdoors or domestic kitchens (Mueller, 2021; McDonnell et al., 2022). They understandably imagined its use only in a contained laboratory with a single intended product.

### Null segregants are organisms with new combinations of genetic material

Null segregants are the last step in some genetic engineering production lines. Null segregants fall within the inclusion clauses of legislative instruments such as Directive 2001/18/EC Article 2(2) when they are organisms with new combinations of genetic material created by techniques of gene technology (Box 1) not excluded or exempted.

Null segregants may routinely carry unintended legacy insertions of DNA contaminating the commercial formulations in which genome editing reagents (e.g., nucleases and/or oligonucleotides) are supplied (Ono et al., 2019), have undetected legacy deletions (Williams, 2022), or other kinds of changes such as base-editing induced point mutations (Wada et al., 2020). The Recombinetics, Inc., cattle episode is illustrative. Even when the eyes of the world were on this company (in part because of boisterous claims of precision in their work), they failed to find thousands of unintended additional nucleotides including antibiotic resistance genes inserted into the cattle genomes (Norris et al., 2020; Solomon, 2020).

Removing null segregants from the GMO regulations requires a predetermination that in the many ways genome editing can be used, a hazard will never arise in null segregants. Deregulation on this basis removes the motivation for technology users to discover the information to assess their products (Agapito-Tenfen et al., 2018; Kawall et al., 2020). It is worth recalling that this was a concern of Fonterra, New Zealand’s largest company and the sixth largest dairy company in the world. It is likely to be a concern for other food system companies.

**FIGURE 1:**
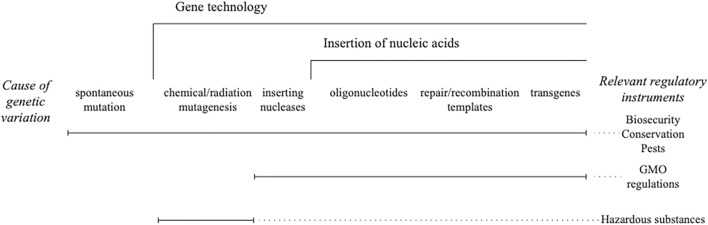
*Genus risk* framework for gene technology. Different legislative instruments are used to control the risk of new combinations of heritable material that are scaled by gene technology.

Taking genome editing of any kind out of the regulated risk genus is an unprecedented step and out of step with all other techniques of gene technology. While chemical and radiation mutagenesis is not usually governed by gene technology (GMO) regulations, it is regulated. The tools have controls in place for access and physical infrastructure for use and disposal. The products have a post release notification that creates liabilities for manufacturers. Having those controls in place made it redundant to include the organisms modified using chemical and radiation mutagens within the GMO regulations, not because they were outside the *genus risk* framework (Figure 1) but because they were already regulated in some fashion (Heinemann et al., 2021).

The aforementioned option is lost if either null segregants or genome editing are placed outside of GMO regulations. Genome editing reagents are commercially available. Guide nucleic acids can be designed using free tools on the internet and ordered at the same time. The scale constraints of earlier techniques ranging from chemical/radiation mutagenesis to synthesis of transgenes, with their need for highly skilled workers in expensive laboratories, are removed. Unfortunately, those who comment on the inconsistency of including genome editing in gene technology regulation when chemical and radiation mutagenesis is not (e.g., Fritsche et al., 2018; Thygesen, 2019; Bartsch et al., 2020) miss this essential point.

The scale of mutagenesis possible through genome editing reagents and methods (Friedrichs et al., 2019; Le Page, 2019; Ahmar et al., 2021) to both professional and lay users, or technical experts in non-expert applications, exceeds what has been anticipated (Monast, 2018). That, however, is just one way contributing to the likelihood of creating a hazard. Gene technology has the ability to tinker with genes, but more importantly to plug into the power of evolution.

Evolution has two ingredients—variation and selection. It is humans that serve as a determinative agent of selection for products of gene technology, including null segregants. As such agents, human institutions and behaviours are also contributors to what and how biologicals can be hazards. In addition, they may also change the severity of harm by altering the scale of exposure.

In his famous essay ‘*Evolution and Tinkering*’ (Jacob, 1977), molecular geneticist F. Jacob reflected on these two parts of the evolutionary process. He besought us in the molecular sciences to not confuse the focus of our techniques, which generate variation by changing nucleotide sequences, with an understanding of their impacts in the world. It is not sufficient in a risk context to only consider whether a biochemical reaction in a test tube is the same as a spontaneous reaction ‘in nature’ when ‘[n]atural selection has no analogy with any aspect of human behavior’ (Jacob, 1977). The latter is indispensable for evaluating the risk trajectory.

Compounding the speed of development is the ability of the ‘engineer’, in Jacob’s sense, to breed large numbers of modified organisms and distribute them globally at unnatural rates unbuffered by stochastic events that lead to an organism’s failure to reproduce. Indeed, the ultimate purpose is to make products at relatively or absolutely large numbers under such protections as monopoly-building intellectual property rights (NRC, 1989; Mueller and Flachs, 2022).

In contrast, gene technology regulations appear more similar to a tinkerer’s (Jacob, 1977) evolutionary outcome than one developed through design. This has led to both inconsistencies and a sense of unfairness, especially for genetic engineers. Unfortunately, the predominant expression of resistance has been to recommend weakened social controls of risk mitigation rather than to develop cohesive and coherent regulatory frameworks. Defining null segregants out of bounds does not keep risk contained.

## Footnotes

1 The authors are grateful to Simon Terry and Stephanie Howard for this insight.

2 Companies and academics with links to companies, most of who would go on to receive the greatest financial rewards from releasing GM plants, comprised at least a third of the membership on the NRC plant panel.
